# Investigations on some indicators in the blood of cattle with orosthenic activity tongue rolling

**DOI:** 10.5455/javar.2023.j685

**Published:** 2023-06-30

**Authors:** Rumen Binev

**Affiliations:** Department of Internal Medicine, Faculty of Veterinary Medicine, Trakia University, Stara Zagora, Bulgaria

**Keywords:** Tongue rolling, hematological indices, cattle

## Abstract

**Objective:**

Hematological studies were carried out in cattle with tongue-rolling hyperactivity in order to establish the etiopathogenetic mechanisms of this behavioral disorder.

**Materials and Methods:**

Cattle were divided into two groups: Group 1 (*n = *12), control animals that did not demonstrate the tongue-rolling orosthenic activity, and Group 2 (*n = *12), cattle that manifested this stereotyped behavior. Blood samples were collected from all cattle twice at 10-day intervals from *Vena jugularis* for analysis of red blood cell counts, hemoglobin (HGB) content, mean corpuscular volume (MCV), mean corpuscular HGB (MCH), mean corpuscular HGB concentration (MCHC), hematocrit (HCT), white blood cell counts (WBC), and differential white cell counts [lymphocytes (LYM), monocytes (MON), and granulocytes (GRAs)]. Some blood biochemical constituents were assayed: calcium, inorganic phosphorus, magnesium, plasma total protein, blood glucose, total bilirubin, urea, creatinine, chlorides, cholesterol, triglycerides, and albumin, as well as the enzyme activities of aspartate amino-transferase (AST), alanine amino-transferase (ALT), alkaline phosphatase (ALP), gamma-glutamyltransferase (γ-GT), lactate dehydrogenase (LDH), and creatine kinase (CK). Blood concentrations of the hormones adrenaline, noradrenaline, cortisol, adrenocorticotropic hormone (ACTH), dopamine, serotonin, free thyroxine, thyroid-stimulating hormone, and trace elements manganese, copper, and iron were determined.

**Results:**

It was found out that the cattle with tongue-rolling hyperactivity did not demonstrate changes in red blood picture (HGB, HCT, MCV, MCH, MCHC), white blood picture (WBC, LYM, MON, GRA), blood biochemistry (Ca, P, Mg, TP, Glu, TB, urea, creatine, Cl, Chole, TG, and Alb), AST, ALT, ALP, γ-GT, LDH, and CK activities, Cu, and Fe levels. In the study, increased concentrations of adrenaline, noradrenaline, cortisol, ACTH, and serotonin were established.

**Conclusion:**

The data demonstrating increased levels of adrenaline, noradrenaline, cortisol, and ACTH confirmed the etiological role of environmental stressors in the emergence of oral hyperactivity. Increased serotonin concentrations in the blood suggest that cattle with the stereotype are probably “happy” with tongue rolling. The lack of changes in blood trace elements manganese, copper, and iron allowed excluding their deficiency as a probable cause in the etiology of the disorder.

## Introduction

Tongue-rolling is considered a locomotor obsessive-compulsive disorder because it is unprovoked, prolonged, and repeated [1–3]. Different forms of conflicting behavior are caused by a feeling of frustration or conflict. After frequent repetition and continuous conflict or frustration, this stereotypy could evolve into “emancipated” (free from dependence, prejudices, and limitations) compared to the animal’s normal behavior and be exaggerated, repetitive, and stable. The term frustration is used in veterinary psychology to denote a state when the animal is motivated to perform a given activity but is restrained from doing it, ending with disappointment. A specific trait of this behavior is that it is manifested in different situations at a decreasing level of excitement [4,5].

Studies on some of the biochemical indicators in cows have been conducted previously [6–8]. The genesis of “tongue rolling” is unclear, but previous suggestions about a deficiency of manganese and other minerals have found little support today. Literature data about the role of some nutritional factors, such as Cu, Zn, Mn, Co, Fe, etc., in the etiology of tongue rolling (play) disorder is rather contradictory, Kirmizigul says. In the view of İssi et al. [9], manganese is an essential trace element for animals, and its deficiency in cattle results in disturbed development, infertility, abortions, joint deformities, abnormalities, and the expression of oral hyperactivities. The authors outlined the association between manganese deficiency and copper deficiency in the disorder’s etiology. As copper is a co-factor for the synthesis of dopamine beta monooxidase [10], it is believed that the low activity of this enzyme has a primary role in the development and diagnosis of tongue rolling disorder [9].

Despite the absence of literature data from an investigation on tongue rolling in cattle supporting the assumption that the animals might experience pleasure, there is an increasing number of reports (especially in men and primates) that a considerable part of behavioral disorders due to deficiency of stimuli (boredom) is performed for pleasure experience. This hypothesis is supported by data from studies of ours in horses demonstrating the locomotor activity “weaving” and showing increased blood concentrations of endorphins (hormones of happiness) during the clinical manifestation of the stereotypy [11].

Investigations performed in 3 farms in the region of Elazig, Turkey, in 15 cattle exhibiting tongue-rolling symptoms [9] reported no significant deviations in the values of hematological parameters from white and red blood pictures [erythrocytes, leukocytes, hemoglobin (HGB), hematocrit (HCT)], concentrations of assayed vitamins (A, C, and β-carotene) and minerals [zinc (Zn), iron (Fe), and cobalt (Co)]. Statistically significant changes were established only in the trace elements copper (Cu) and manganese (Mn). Based on these data, the authors outlined the role of mineral deficiency in the etiology of disease and the need for prophylactic dietary supplementation of animals with copper and manganese.

On the contrary, Okatan et al. [12] affirmed that trace elements had no role in the etiology of oral stereotypy in cattle. This conclusion was made after studying 40 animals manifesting tongue rolling in another region of Turkey, Kayseri, with respect to blood serum concentrations of copper, zinc, manganese, iron, chrome, and nickel. The results showed no statistically significant difference in the levels of these trace elements between healthy and diseased cattle.

Previous studies established the causes, prevalence, age- and sex-related features, and frequency and 24-h pattern of clinical signs accompanying tongue-rolling stereotypy in cattle [13]. It was found that the causes of tongue-rolling hyperactivity in cattle were food deprivation and boredom due to a deficiency of environmental stimuli. The studied stereotypy was observed in cattle in different age groups: suckling and weaned calves, as well as heifers, including pregnant ones. The tongue play activity was no longer exhibited by heifers after calving and moving into the basic herd of lactating cows. The highest intensity (up to 80%) of this orosthenic syndrome manifested before the morning feeding, between 9.00 and 12.00 a.m. Most commonly, tongue-rolling activity was found in heifers between 13 and 25 months of age. The studied oral stereotypy was not accompanied by changes in measured clinical parameters (rectal body temperature, pulse, respiratory rates, rumen movements, or other activities, e.g., rumination, eructation, appetite, sentience, locomotion, etc.).

## Material and Methods

### Ethical statement

The study was performed by following the Institutional Animal Ethical Committee of the Trakia University, Bulgaria, for collecting blood from animals and according to National Ordinance No. 20 of November 2012 on the minimum requirements for the protection and humane treatment of animals.

### Animals

The studies were carried out on five cattle farms in different regions of the country and on patients of the Farm Animal Clinic at the Faculty of Veterinary Medicine, Trakia University. Out of all 1,103 surveyed cattle, 48 demonstrated tongue-rolling orosthenic activity.

### Collection of samples

Two groups were formed: Group 1 (*n = *12), healthy controls, and Group 2 (*n = *12), cattle suffering from tongue rolling. Blood was sampled from all animals from *Vena jugularis* for analysis of red blood cell counts (T/l), HGB content (gm/l), mean corpuscular volume (MCV, fl), mean corpuscular HGB (MCH, pg), mean corpuscular HGB concentration (MCHC, gm/l), HCT (l/l), white blood cell counts (gm/l), differential white cell counts (DWC)—lymphocytes (LYM, %), monocytes (MON, %), and granulocytes (GRA, %) (in absolute and percentage values), on an automated hematological analyzer BC-5000 Vet, Mindray, China.

 Blood biochemical parameters (calcium, inorganic phosphorus, magnesium, total protein, glucose, total bilirubin, urea, creatinine, chlorides, cholesterol, triglycerides, and albumin) and enzyme activity of aspartate aminotransferase (AST, U/l), alanine aminotransferase (ALT, U/l), alkaline phosphatase (ALP, U/l), gamma-glutamyltransferase (γ-GT, U/l), lactate dehydrogenase (LDH, U/l), and creatine kinase (CK, U/l) was assayed on an automated biochemical analyzer BS-120, Mindray, China.

The concentrations of adrenaline (ng/l), noradrenaline (ng/l), cortisol (nmol/l), adrenocorticotropic hormone (ACTH, nmol/l), dopamine (ng/l), and serotonin (ng/l) were analyzed by high-performance liquid chromatography by Agilent in MVZ Labor Dr. Limbach, Heidelberg, Germany.

The levels of thyroid-stimulating hormone (mIU/l) and free thyroxine (FT_4_, nmol/l) in blood plasma were assayed via chemiluminescence immunoassay (*CLIA* chemiluminescence immunoassays on an automated biochemical analyzer, Adviva Centaur XP, Siemens, Germany).

The trace element manganese in the blood (Mn, μg/l) was determined via inductive coupled plasma mass spectrometry in MVZ Labor Dr. Limbach, Heidelberg, Germany.

Copper levels (Cu, μmol/l) were assayed through atomic absorption spectrometry in MVZ Labor “Dr. Limbacch,” Heidelberg, Germany, and concentrations of iron (Fe, μmol/l)—using the direct method (Ferene), on an automated chemical analyzer Architect c8000, Abbott, Switzerland.

**Table 1.** Changes in the values of erythrocytes, HGB, MCV, MCH, MCHC, HCT, thrombocytes, leukocytes, LYMs, MON, and GRAs (absolute counts and percentage) in cattle: control (Group 1) and demonstrating “tongue rolling” activity (Group 2).

Group	Erythrocytes (10^12^/l)	HGB (gm/l)	Haematocrit (%)	MCV (fl)
1	7 ± 0.76	100.73 ± 9.10	25.89 ± 4.15	62.8 ± 7.2
2	6.48 ± 0.95^ns^	104.20 ± 11.23^ns^	24.65 ± 3.37^ns^	70.4 ± 8.8^ns^
Group	MCH (pg)	MCHC (gm/l)	Thrombocytes (10^9^/l)	
1	14.74 ± 2.12	382.80 ± 11.55	435.60 ± 42.30	
2	14.94 ± 2.02^ns^	384.78 ± 12.41^ns^	362.67 ± 28.32^ns^	
Group	Leukocytes (10^9^/l)	LYMs (10^9^/l)	GRAs (10^9^/l)	MONs (10^9^/l)
1	10.31 ± 0.81	5.36 ± 0.43	2.52±0.24	0.92 ± 0.066
2	8.87 ± 0.33^ns^	6.35 ± 0.40^ns^	2.64±0.19^ns^	0.81 ± 0.058^ns^
Group	LYMs (%)	MONs (%)	GRAs (%)	
1	66.63 ± 5.86	7.96 ± 0.59	25.89 ± 1.71	
2	65.34 ± 4.19^ns^	8.01 ± 0.67^ns^	24.65 ± 1.87^ns^	

Difference from the control group; n.s.: non-significant; MCV: mean corpuscular volume; MCH: mean corpuscular hemoglobin; MCHC: mean corpuscular hemoglobin concentration.

### Statistical analysis

All data were processed using statistical software (Statistica 6.0 for Windows, StatSoft Inc., USA, 1993). The differences between groups were evaluated by means of Student‘s tests at a level of significance of *p *< 0.05.

## Results

### Red and white blood pictures

The values of the red blood picture parameters are presented in Table 1. The amounts of all measured indices [erythrocyte counts, HGB content, MCV, MCH, MCHC, HCT, thrombocyte counts, and DWCs (leukocytes, LYMs, MONs, and GRAs) as absolute values and percentages] in the blood of cattle manifesting tongue-rolling stereotypy were insignificantly different from those in the control cattle group (*p *> 0.05).

Parameters of the white blood cell picture showed no statistically significant alterations (*p *> 0.05) in total leukocyte counts, as well as in both absolute values and percentages of LYMs, MONs, and GRAs in control cattle (Group 1) compared to those exhibiting the abnormal tongue rolling oral hyperactivity.

### Concentrations of calcium, inorganic phosphorus, magnesium, plasma total protein, blood glucose, total bilirubin, urea, creatinine, chlorides, cholesterol, triglycerides, and albumin

The blood values of these analytes in control and tongue-rolling cattle are shown in Table 2. The analysis of blood concentrations of calcium, inorganic phosphorus, magnesium, plasma total protein, blood glucose, total bilirubin, urea, creatinine, chlorides, cholesterol, triglycerides, and albumin showed no difference (*p* > 0.05) between the group of tongue rolling cattle and the control group.

### Enzyme activity of AST, ALTALP, γ-GT, LDH, and CK

The changes in blood activities of AST, ALT, ALP, γ-GT, LDH, and CK in control cattle and animals manifesting tongue-rolling oral hyperactivity are shown in Table 3.

The enzyme activities of AST, ALT, ALP, *γ-GT*, LDH, and CK in the blood of tongue-rolling cattle did not differ from respective measurements in control animals (*p* > 0.05).

### Concentrations of adrenaline, noradrenaline, cortisol, ACTH, serotonin, dopamine, thyroid-stimulating hormone, FT_4_, and concentrations of trace elements manganese, copper, and iron

The results from analysis of changes in adrenaline, noradrenaline, cortisol, ACTH, serotonin, dopamine, thyroid-stimulating hormone, FT_4_, and concentrations of trace elements (manganese, copper, and iron) in both control and tongue-rolling cattle are shown in Table 4.

**Table 2.** Changes in the concentrations of calcium, inorganic phosphorus, magnesium, plasma total protein, blood glucose, total bilirubin, urea, creatinine, chlorides, cholesterol, triglycerides, and albumin in cattle: control (Group 1) and demonstrating “tongue rolling” activity (Group 2).

Group	Calcium (mmol/l)	Inorganic phosphorus (mmol/l)	Magnesium (mmol/l)	Total protein (gm/l)
1	2.954 ± 0.11	1.88 ± 0.27	0.88 ± 0.12	77.69 ± 5.59
2	2.97 ± 0.20^ns^	1.91 ± 0.17^ns^	0.93 ± 0.08^ns^	74.11 ± 6.65^ns^
Group	Blood glucose (mmol/l)	Total bilirubin (μmol/l)	Urea (mmol/l)	Creatinine (μmol/l)
1	4.00 ± 0.29	5.22 ± 0.27	1.92 ± 0.21	96.13 ± 3.87
2	3.95 ± 0.13^ns^	6.74 ± 0.39^ns^	2.07 ± 0.21^ns^	98.73 ± 4.84^ns^
Group	Chlorides (mmol/l)	Cholesterol (mmol/l)	Triglycerides (mmol/l)	Albumin (gm/l)
1	100.60 ± 4.53	2.86 ± 0.14	0.20 ± 0.03	32.95 ± 2.62
2	98.73 ± 5.84^ns^	2.87 ± 0.25^ns^	0.24 ± 0.02^ns^	34.78 ± 1.81^ns^

Difference from the control group; n.s.: non-significant.

**Table 3.** Changes in enzyme activities of AST, ALT, ALP, γ-GT, LDH, and CK in cattle: control (Group 1) and demonstrating “tongue rolling” activity (Group 2).

Group	AST (U/l)	ALT (U/l)	ALP (U/l)
1	87.80 ± 4.98	28.13 ± 1.79	181.87 ± 21.11
2	98.20 ± 5.16^ns^	19.67 ± 1.88^ns^	217.40 ± 19.81^ns^
Group	γ-GT (U/l)	LDH (U/l)	CK (U/l)
1	17.73 ± 2.21	1926.9 ± 122.65	228 ± 16.27
2	11.78 ± 2.65^ns^	1568.3 ± 18.99^ns^	150.40 ± 10.21^ns^

Difference from the control group; n.s.: non-significant.

The data showed statistically significant differences between groups with respect to levels of adrenaline, noradrenaline, cortisol, ACTH, and serotonin.

Cattle exhibiting tongue-rolling oral hyperactivity had higher concentrations of enumerated hormones as follows: 26.93 ± 2.30 ng/l, 66.34 ± 4.44 ng/l, 54.37 ± 5.51 nmol/l, 236.67 ± 17.74 nmol/l, and 353.87 ± 30.8 ng/ml, versus respective average control values: 20.05 ± 2.21 ng/l (*p *< 0.05), 52.93 ± 3.29 ng/l (*p *< 0.05), 28.37 ± 3.816 nmol/l (*p* < 0.01), 96.61 ± 7.81 nmol/l (*p *< 0.01) and 285.17 ± 23.98 ng/ml (*p *< 0.05).

No significant differences were found in the concentrations of dopamine, thyroid-stimulating hormone, FT_4_, and levels of manganese, copper, and iron between control and tongue-rolling cattle (*p *> 0.05).

## Discussion

The present studies aimed at evaluating the parameters of red blood picture (erythrocyte counts, HGB content, MCV, MCH, MCHC, HCT, and thrombocyte counts) and white blood picture (leukocytes, absolute values, and percentages of LYMs, MONs, and GRAs) showed that they were insignificant changes in tongue-rolling cattle as compared to healthy controls. Our data are in line with those reported by Wilson et al. [14].

**Table 4.** Changes in the hormonal activity of adrenaline, noradrenaline, cortisol, ACTH, serotonin, dopamine, thyroid-stimulating hormone, FT_4_ in cattle and concentrations of trace elements manganese, copper, and iron: control (Group 1) and demonstrating “tongue rolling” activity (Group 2).

Group	Adrenaline (ng/l)	Noradrenaline (ng/l)	Cortisol (nmol/l)	ACTH (nmol/l)
1	20.05 ± 2.21	52.93 ± 3.29	28.37 ± 3.816	96.61±7.81
2	26.93 ± 2.30^*^	66.34 ± 4.44^*^	54.37 ± 5.51^**^	236.67±17.74^**^
Group	Serotonin (ng/ml)	TSH (mIU/l)	FT_4_ (nmol/l)	Dopamine (ng/l)
1	285.17 ± 23.98	0.013 ± 0.002	29.33 ± 2.81	18.32 ± 4.62
2	353.87 ± 30.8^*^	0.012 ± 0.0017^n.s^	28.34 ± 3.77^ns^	17.72 ± 4.11^ns^
Group	Manganese (μg/l)	Copper (μmol/l)		Iron (μmol/l)
1	6.48 ± 0.46	11.19 ± 1.04		22.72 ± 1.23
2	6.29 ± 0.53^ns^	11.15 ± 0.94^ns^		22.62 ± 1.28^ns^

^*^p < 0.05; ^**^p < 0.01.

Difference from the control group; n.s.: non significant; ACTH: adrenocorticotropic hormone; TSH: thyroid-stimulating hormone; FT_4_: free thyroxine.

No significant differences were also identified for some blood biochemical parameters (calcium, inorganic phosphorus, magnesium, chlorides, total bilirubin, total protein, blood glucose, urea, creatinine, cholesterol, triglycerides, and albumin) between controls and cattle with tongue rolling stereotypy.

A similar tendency was found in the blood enzyme activities of AST, ALT, ALP, γ-GTLDH, and CK in both studied groups of cattle (normal and affected).

Our data evidencing no deviations in studied biochemical blood parameters and enzyme constellations are somewhat comparable with those reported by Prodanović et al. [6,7] in high-yielding dairy cows in Serbia reared under an intensive production system. Unlike us, the authors have found substantially lower blood concentrations of glucose, proteins, albumin, urea, and magnesium and increased total bilirubin and AST activity. These changes were, however, registered only in cows from the group formed 40 days postpartum. In our opinion, this period coincides with the most intensive lactation and hence the highest loss of nutrients (carbohydrates, proteins, fats, minerals, vitamins, etc.) with milk. We disagree with the statement about the higher prevalence of tongue “play” in this group of cows (40 days postpartum) attributed to lower levels of glucose, protein, albumin, and urea and with the hypothesis that reduced magnesium concentrations could be involved in the etiopathogenesis of this oral hyperactivity. Another fact in support of our disagreement with previous reports [6–8] is the fact that in our studies, oral hyperactivity was observed in cattle from 2 months of age until calving but never in parturient cows.

In our study, differences between healthy cattle and tongue-rolling animals were identified only in the blood concentrations of some hormones. The most pronounced changes between control and tongue-rolling cattle were detected in concentrations of the so-called stress hormones: adrenaline, noradrenaline, cortisol, and ACTH. The results are aligned with previous reports [15–17], where different age groups of cattle have been considered.

The absence or restricted possibility of locomotion, feeding, or evenness of the environment could result in serious frustration, fairly sufficient to induce almost constant stress in animals, which causes adrenal gland hyperfunction correlating with the increased levels of studied hormones [17,18]. This fact indicates that tongue rolling oral hyperactivity is a sign of a compromised “social” environment causing “dissatisfaction” from the inability or restricted expression of the natural behavior [15,16,19,20].

Simultaneously with increased stress hormone levels, we detected increased blood serotonin concentrations as well. This fact suggested the hypothesis that cattle manifesting tongue-rolling stereotypy are likely to experience pleasure from this abnormal oral hyperactivity. It is assumed that the increase in serotonin is negatively proportional to the amount of dopamine [19,20]. In the present studies, however, statistically significant changes in the levels of this hormone, as well as in the concentrations of thyroid-stimulating hormone and FT_4_, were not found.

The analysis of some blood trace minerals (manganese, copper, and iron) in tongue-rolling cattle showed no significant differences versus controls. The findings of our study are similar to the results on the mineral profile of cattle with this oral stereotypy from different regions in Turkey [12] for manganese, copper, and iron and the results described previously [9,21], but only with regard to blood iron. The observed lack of differences in blood copper and manganese differed from the report of İssi et al. [9], affirming that these two trace minerals were decreased in the blood of tongue-rolling cattle. The data about blood trace minerals in tongue-rolling cattle support the assumption [12] that these minerals played no etiological role in the emergence of this bovine oral stereotypy.

## Conclusion

Results from blood laboratory analyses showed no changes in the red blood picture (erythrocyte counts, HGB content, MCV, MCH, MCHC, HCT, and thrombocyte counts) or white blood picture (leukocytes, absolute values, and percentages of LYMs, MONs, and GRAs). No significant differences in blood biochemical parameters (calcium, inorganic phosphorus, magnesium, chlorides, total bilirubin, total protein, blood glucose, urea, creatinine, cholesterol, triglycerides, and albumin), and blood enzymes (AST, ALT, ALP, *γ-GT*, LDH, and CK) were identified between controls and cattle with tongue-rolling oral hyperactivity. Our data demonstrating increased levels of adrenaline, noradrenaline, cortisol, and ACTH confirmed the etiological role of environmental stressors in the emergence of oral hyperactivity. Increased serotonin concentrations in the blood suggested that cattle with the stereotype are probably „happy“ with tongue rolling. The lack of changes in blood trace elements manganese, copper, and iron allowed excluding their deficiency as a probable cause in the etiology of the disorder. From a therapeutical and prophylactic point of view, environmental stimuli should be enriched in order to increase the involvement of animals and restrict the states of „expectation“ and „boredom.“ This could be achieved by increasing feeding frequency, including non-ground roughage (hay, straw, etc.), and providing conditions for grazing and higher locomotor activity.

## List of abbreviations

HGB, hemoglobin; MCV, mean corpuscular volume; MCH, mean corpuscular hemoglobin; MCHC, mean corpuscular hemoglobin concentration; HCT, hematocrit; WBC, white blood cell counts; DWC, differential white cell counts; LYM, lymphocytes; MON, monocytes; GRA, granulocytes; AST, aspartate aminotransferase; ALT, alanine aminotransferase; ALP, alkaline phosphatase; γ-GT, gamma-glutamyltransferase; LDH, lactate dehydrogenase; CK, creatine kinase; ACTH, adrenocorticotropic hormone; FT_4_, free thyroxine; Mn, manganese; Cu, Copper; Fe, iron.
